# Acute cystitis and subsequent risk of urogenital cancer: a national cohort study from Sweden

**DOI:** 10.1136/bmjph-2024-002495

**Published:** 2025-09-16

**Authors:** Filip Jansåker, Xinjun Li, Kristina Sundquist

**Affiliations:** 1 Center for Primary Health Care Research, Department of Clinical Sciences Malmö, Lund University, Lund, Sweden; 2 University Clinic Primary Care Skåne, Skåne University Hospital, Malmö, Sweden; 3 Department of Clinical Microbiology, Copenhagen University Hospital Rigshospitalet, Copenhagen, Denmark

**Keywords:** Epidemiology, Risk Assessment, Public Health, Epidemiologic Measurements

## Abstract

**Introduction:**

This study aimed to examine the subsequent risks of urogenital cancers in men and women aged ≥50 years diagnosed with acute cystitis.

**Methods:**

A Swedish nationwide cohort study was conducted that included primary healthcare data. The total population consisted of 1 668 371 men and 1 889 211 women; of these, 177 736 men and 427 821 women were diagnosed with acute cystitis (first event) during 1997–2018 (91.3% in primary healthcare settings). The outcome was urogenital cancer in men and women with cystitis compared with the total population, measured as standardised incidence ratios (SIRs) and excess rates per 10 000 person-years.

**Results:**

A total of 199 144 men and 57 882 women were diagnosed with urogenital cancer (24 137 subsequently to cystitis). The most common cancers were prostate and bladder cancer. The urogenital cancer risks were elevated across all age groups of men and women with cystitis. The risks were highest within 3 months of follow-up but persisted over several years for most cancers. The risks within 3 months of follow-up were as follows: for bladder cancer, the SIR was 33.69 (95% CI 32.02 to 35.43) in men and 30.00 (28.02 to 32.09) in women, corresponding to 483.72 and 96.00 excess cancer rates per 10 000 person-years, respectively. For prostate cancer, the SIR was 7.05 (6.74 to 7.37) and the excess cancer rate was 550.88 per 10 000 person-years; between 3 and 12 months of follow-up, the excess rate was 104.89 per 10 000 person-years. For kidney cancer, the SIR was 11.28 for men and 7.72 for women. For gynaecological cancers, SIRs were between 4 and 8. Some interactions were observed between sex and cystitis in relation to urogenital cancer risks.

**Conclusion:**

Acute cystitis can precede urogenital cancers in men and women aged ≥50 years. The increased risks were particularly high within 3 months after the acute cystitis event and persisted for several years.

**WHAT IS ALREADY KNOWN ON THIS TOPIC**

- Acute cystitis is a common infection of the lower urinary tract, mostly presenting in primary healthcare settings.
- Acute cystitis might be a clinical marker for urogenital cancer, but the existing evidence is sparse and does not include nationwide data from primary healthcare settings.

**WHAT THIS STUDY ADDS**

- In adults aged 50 years and older, acute cystitis was primarily diagnosed in primary healthcare and found to precede urogenital cancers.
- The urogenital cancer risks were particularly high within the first 3 months after an acute cystitis event, especially for prostate and bladder cancer, and persisted for many years for most cancers.

**HOW THIS STUDY MIGHT AFFECT RESEARCH, PRACTICE OR POLICY**

- Acute cystitis may act as a useful predictor of urogenital cancer in men and women aged 50 years and older.

## Introduction

 Urinary tract infections (UTIs) are highly prevalent, often recurring acute infections in the population, affecting over 150 million people each year worldwide. UTIs often result in frequent patient visits to primary healthcare.^1^,^4^ The clinical picture of UTI is heterogeneous, spanning from the most common and least severe form, acute cystitis (lower UTI), to pyelonephritis (upper UTI) and urosepsis.^4^,^7^ The lifetime prevalence of UTI is 50–60% in women^1^ and 13–14% in men, where men account for approximately 20% of all UTI cases.^8^ With the exception of a spike in young women,^5^ the incidence rates of UTI increase with age in both sexes.^1 3^ In postmenopausal w-men, the 12 month prevalence is about 10%,^1^ and in elderly men and women, the rates are comparable.^9^ This age-related increase in UTIs, particularly in adults aged 50 years and older, is mainly because of the higher prevalence of risk factors, including malignant diseases^7 10 11^ that compromise the immune defence in the urinary system.

Urogenital cancers are associated with significant morbidity and mortality worldwide^12^ and an increased risk of UTIs.^3 7^ A population-based cohort study showed that a hospital-based diagnosis of pyelonephritis could be a clinical marker of urogenital cancer in adults.^13^ Although studies have shown that acute cystitis might also be linked to an increased risk of urogenital cancers,^14^,^16^ the evidence is limited to studies that did not include data from primary healthcare settings, which is where most acute cystitis cases are diagnosed.^1 3 4^ We are unaware of any large-scale population-based studies examining the subsequent risk of urogenital cancers in men and women with acute cystitis that include primary healthcare data. Given that acute cystitis is the most common form of UTI,^4^,^7^ and as many urogenital cancers may present with overlapping symptoms^5 7^ in primary healthcare settings before the cancer diagnosis,^17 18^ it is possible that acute cystitis might precede urogenital cancer. Such knowledge could have important clinical and public health implications.

This nationwide study, based on primary healthcare and national register data, aimed to assess the subsequent risks of the most common urogenital cancers among men and women aged 50 years and older diagnosed with acute cystitis, stratified by different follow-up periods and age groups.

## Materials and methods

### Design and setting

We conducted a national cohort study of adults aged 50 years and older in Sweden, which had an estimated 8.8 million residents at the beginning of the study period (1 January 1997) and 10.3 million residents at the end of the study period (31 December 2018).^19^ Sweden has a universal tax-financed healthcare service with the goal of providing healthcare on equal terms for the entire population.^20^ At birth or on immigration, all people residing in Sweden are assigned a unique 10-digit personal identification number. This number is used at all healthcare contacts and for the collection of data by public authorities for national registers, thus enabling accurate linkage between medical data and public registries.^21^ Several comprehensive registers and primary healthcare data sources were used in this study, containing individual-level information on all people living in Sweden, including age, sex, socioeconomic status, region of residence, cancer diagnoses and date of cancer diagnoses, hospital diagnoses and dates of hospital admissions, primary healthcare diagnoses and dates of primary healthcare contacts, date of emigration, as well as date and cause of death.^22^,^24^

### Data sources

The Total Population Register (1968–2018), which is managed by the Swedish governmental authority Statistics Sweden (SCB), includes data on the whole Swedish population, including death, emigration, immigration and sociodemographic variables.^24^ The Swedish National Patient Register, managed by the Swedish National Board of Health and Welfare (in Swedish: Socialstyrelsen), includes inpatient (1964–2018) and outpatient specialist care medical diagnoses (2001–2018).^23^ During the study period, diagnosisnose codes were collected according to the 10th edition of the International Classification of Diseases (ICD-10). The Swedish National Cancer Register (1958–2018, Socialstyrelsen) includes all cancer diagnoses (ICD-7 coded) in Sweden and is used for monitoring cancer incidence and survival rates.^22^ Approximately 60 000 unique cancer cases are reported to this register annually. All cancer cases are assigned a date of clinical diagnosis. The population-based primary healthcare data (1997–2018) used in this study were collected from 20 out of 21 administrative healthcare regions in Sweden. These data include individual-level healthcare information (ICD-10 coded) based on visits to primary healthcare centres in Sweden. The population-based coverage of these data varied over time and region throughout the study period, depending on the time of digitalisation of the patient records in a certain region. In 2015, the register contained 72% of Sweden’s population, with approximately 208 million registrations from 7.4 million individuals,^25^ and around 90% of the population at the end of the study period.^4^

### Study population

Age was restricted to men and women ≥50 years of age because urogenital cancer risk in the younger population is low.^11 13 26^ After excluding 34 358 people with previously diagnosed urogenital cancer (3 year wash-out period), the study included a total of 3 557 582 adults aged ≥50 years on 1 January 1997 (onlinesupplemental information Table S1). Individuals from this population were included in the cystitis cohort (n=6 05 557) on their first recorded diagnosis of cystitis during the study period. The cystitis diagnoses were identified in primary healthcare data and the National Patient Register from 1 January 1997 to 31 December 2018. Both primary and secondary ICD-10 coded diagnoses of cystitis (N30) were considered.^4^ We did not consider cystitis episodes occurring subsequent to a urogenital cancer, diagnosis nor did we consider subtypes of cystitis that were not classified as acute infective cystitis (that is, N301, N302, N303, N304 and N308). We also assessed the setting of cystitis diagnosis, that is, whether the infection was diagnosed in outpatient specialist care or primary healthcare settings (online supplemental table S2).

### Urogenital cancers

Data on cancer incidence were collected from the Swedish Cancer Register.^22^ The outcomes (ICD-7 codes, online supplemental table S3) were cancers in the urinary organs: urinary bladder and other (‘181’, in this paper called bladder cancer); cervix uteri (‘171’, in this paper called cervical cancer); corpus uteri and uterus (‘172’ and ‘174’, in this paper called endometrial cancer); prostate (‘177’, in this paper called prostate cancer); kidney (‘180’ including renal pelvis ‘1801’, in this paper called kidney cancer); and ovary, tube and broad ligament’ (‘175’, in this paper called ovarian cancer).

### Covariates

From the Total Population Register, we collected data on sociodemographic factors at baseline used in the adjustments. We considered region of residence and education level (proxy for socioeconomic status) as potential confounders based on previous research.^4 27^ Education level was categorised into two different categories based on the duration of school years attended: <12 years or ≥12 years. Region of residence was categorised into residing in large cities (Malmö, Gothenburg or Stockholm) or outside large cities. We also considered country of origin, categorised into born in or outside Sweden, as a potential confounder based on previous research.^4 28^ From the National Patient Register and primary healthcare data, we collected data on diagnosed conditions (ICD-10 codes, 1997–2018) known to affect the risk of acute cystitis and cancers (in this paper called comorbidities). Diabetes mellitus, immunodeficiency dsorders, and nephritic/nephrotic syndrome were considered potential confounders for all urogenital cancers.^29^,^33^ Additionally, urolithiasis was considered a confounder for urinary tract cancers, non-malignant prostate diseases (benign prostate hyperplasia and prostatitis) were considered confounders for prostate cancer, and bacterial vaginosis was considered a confounder for cervical cancer.^34^,^36^

### Statistical analysis

We calculated the distribution of baseline characteristics of the total population, individuals diagnosed with urogenital cancer and the cystitis cohort at the time of the cystitis diagnosis. These characteristics included mean age (SD), age category (50–59, 60–69, 70–79 and ≥80 years), sex (male/female), sociodemographic factors (education level, region of residency and country of origin) and comorbidities. We also calculated the cumulative incidence of cystitis and urogenital cancer in the total population for the whole follow-up period (1997–2018).

Each individual was followed from the date of the first diagnosis of cystitis (starting on 1 January 1997) until the date of a first cancer diagnosis, emigration, death or end of study period (31 December 2018), whichever came first. We calculated the total follow-up time in person-years and the mean (SD) follow-up time for the total population and cystitis cohort. Standardised incidence ratios (SIRs) were calculated to compare the relative risk of urogenital cancers in the cystitis cohort with those without cystitis in the total population. The urogenital cancer rates were calculated by combining data from the Total Population Register and the National Cancer Register. SIRs were calculated as the ratio of observed (O) number of cancer cases in the cystitis cohort to expected (E*) number of cancer cases in the total population using indirect standardisation methods.^37^ All calculations were standardised by age (10-year age groups), sex (male and female), time period (5-year period groups) and other covariates. We categorised the follow-up period as 0 to<3 months, 3 to 12 months, >1 to 5 years, and >5 years. Excess rates of urogenital cancers per 10 000 person-years are presented in the tables together with the SIR for each follow-up period. The excess cancer rates per 10 000 person-years were calculated by comparing the observed (O) subsequent cancer cases in men and women with cystitis and the expected (E*) cancer cases divided by the total follow-up time.

The incidence rates (per 10 000 person-years) of cancer in those with and without cystitis were plotted by age. The cancer risks were assessed for men and women separately, with the exception of the rates for bladder and kidney cancers that were plotted for both sexes combined. SIRs were also calculated for different age groups to examine whether the associations varied between different age groups. Interaction tests were also conducted to assess whether the association between urinary tract cancers (bladder and kidney cancer) differed between the sexes. The 95% CIs of the SIRs were calculated assuming a Poisson distribution. The formulas used for calculating the SIRs, 95% CIs of the SIRs and excess cancer rates are included in the online supplemental information. All of the analyses were performed using SAS software version 9.4 (SAS Institute, Cary, NC, USA).

### Patient and public involvement

Although we did not involve patients or members of the public in the study design, analyses, interpretation of results or dissemination of results, the concept of this study emerged from observations in the treatment and care of patients in primary healthcare settings.

### Dissemination to participants and related patient and public communities

Due to the nationwide design and pseudonymisation of data, dissemination of the results to the study participants is not possible. We intend to disseminate the findings of this study to the general public after publication of the results, both through conventional media outreach, such as press releases by the research institutions of the contributing authors, social media outlets and public oral presentations.

## Results

The study included 3 557 582 adults (46.9% men and 53.1% women) aged ≥50 years without a prior diagnosis of urogenital cancer. The mean (SD) age at baseline was 65.8 (±11.6) years and the total follow-up was 55 877 745 person-years, with a mean individual follow-up time of 15.6 (±7.3) years.

### Cystitis cohort

Table 1 shows an overall cumulative incidence of cystitis of 17.0 (95% CI 16.9 to 17.1) during the study period (1997–2018). The cystitis cohort comprised 605 557 people, most of whom were women (70.6%, n=427 821). The total follow-up of the cystitis cohort was 4 027 518 person-years, with a mean (SD) individual follow-up time of 6.7 years (±4.9). The mean age at cystitis diagnosis was 75.9 years (±9.2) and the majority (91.3%, n=552 674) of the cystitis events were diagnosed in primary healthcare settings (online supplemental table S2).

**Table 1:** Number of cases and cumulative incidences of acute cystitis and urogenital cancer in adults aged ≥50 years (n=3 557 582) during the follow-up period (1997–2018)

	Acute cystitis		Urogenital cancers	
	No of cases (%)	Cumulative incidence, % (95% CI)	No of cases (%)	Cumulative incidence, % (95% CI)
Total	605 557	17.0 (16.9 to 17.1)	257 026	7.2 (7.1 to 7.3)
Age groups				
50–59	18 884 (3.1)	1.4 (1.2 to 1.6)	10 738 (4.2)	0.8 (0.6 to 1.0)
60–69	140 861 (23.3)	15.4 (15.2 to 15.6)	72 259 (28.1)	7.9 (7.7 to 8.1)
70–79	226 450 (37.4)	28.3 (28.1 to 28.5)	110 623 (43.0)	13.8 (13.6 to 14.0)
≥80	219 362 (36.2)	43.2 (43.0 to 43.4)	63 406 (24.7)	12.5 (12.2 to 12.7)
Sex				
Male	177 736 (29.4)	10.7 (10.5 to 10.8)	199 144 (77.5)	11.9 (11.8 to 12.1)
Female	427 821 (70.6)	22.6 (22.5 to 22.8)	57 882 (22.5)	3.1 (2.9 to 3.2)
Educational level				
<12 years	437 262 (72.2)	16.8 (16.7 to 16.9)	170 130 (66.2)	6.5 (6.4 to 6.7)
≥12 years	168 295 (27.8)	17.6 (17.4 to 17.8)	86 896 (33.8)	9.1 (8.9 to 9.3)
Region of residence				
Large cities	344 971 (57.0)	17.8 (17.7 to 18.0)	125 920 (49.0)	6.5 (6.4 to 6.7)
Other	260 586 (43.0)	16.0 (15.9 to 16.2)	131 106 (51.0)	8.1 (7.9 to 8.2)
Country of origin				
Born in Sweden	535 709 (88.5)	17.6 (17.5 to 17.7)	233 494 (90.8)	7.7 (7.6 to 7.8)
Born outside Sweden	69 848 (11.5)	13.6 (13.3 to 13.8)	23 532 (9.2)	4.6 (4.3 to 4.8)
Comorbidities				
Diabetes mellitus	133 908 (22.1)	26.3 (26.0 to 26.5)	46 965 (18.3)	9.2 (8.9 to 9.5)
Immunodeficiency disorders	3035 (0.5)	28.4 (26.8 to 30.0)	967 (0.4)	9.1 (7.2 to 10.9)
Nephritic or nephrotic syndrome	31 652 (5.2)	27.6 (27.1 to 28.1)	14 091 (5.5)	12.3 (11.7 to 12.8)
Urolithiasis	36 092 (6.0)	33.5 (33.0 to 34.0)	16 685 (6.5)	15.5 (14.9 to 16.0)
Non-malignant prostate diseases	96 911 (16.0)	25.6 (25.4 to 25.9)	70 574 (27.5)	18.7 (18.4 to 19.0)
Bacterial vaginosis	5164 (0.9)	52.0 (50.7 to 53.4)	488 (0.2)	4.9 (3.0 to 6.8)

Acute cystitis cases were measured as first diagnosis during the study period and diagnoses occurring subsequent to a urogenital cancer diagnosis were not measured. Urogenital cancer cases were measured as the first diagnosis during the study period in those not previously diagnosed with urogenital cancer. Non-malignant prostate diseases include benign prostate hyperplasia and prostatitis. Sociodemographic characteristics and comorbidities of the total population at baseline are presented in online supplemental table S1. The proportion of men diagnosed with non-malignant prostate diseases was 54.5% in the cystitis cohort and 35.4% in those with urogenital cancer. The proportion of women diagnosed with bacterial vaginosis was 1.2% in the cystitis cohort and 0.8% in those diagnosed with urogenital cancer.

### Urogenital cancer

Table 1 shows that the overall cumulative incidence of urogenital cancer was 7.2 (95% CI 7.1 to 7.3) during the study period, resulting in a total of 257 026 people diagnosed with urogenital cancer, most of whom were men (77.5%, n=199 144). The mean (SD) age at cancer diagnosis was 73.52 years (±8.26) and prostate cancer was the most common cancer type (61.8%), followed by bladder (16.4%) and endometrial cancers (9.6%) (online supplemental table S3). Cystitis preceded cancer diagnosis in a total of 24 137 people (9.4% of all those diagnosed with cancer during the study period). Of these people, the mean age at cancer diagnosis was 76.90 years (±7.72) and prostate cancer was the most common cancer type (39.5%), followed by bladder (31.6%) and endometrial (13.8%) cancers. Online supplemental figures S1–S6 display the incidence rates per 10 000 person-years of urogenital cancers in the study participants with and without cystitis; the incidence rates were, in general, higher in those with cystitis across all age groups.

### Urinary tract cancers

For bladder cancer, the SIR was 3.50 (95% CI 3.40 to 3.60) in men with cystitis (table 2), corresponding to an excess cancer rate of 40.05 (39.99 to 40.11) per 10 000 person-years during follow-up. For women (table 3), the corresponding SIR was 3.26 (95% CI 3.15 to 3.37) and the excess cancer rate was 7.77 (7.70 to 7.84) per 10 000 person-years. The risks were particularly high within 3 months of follow-up, with an SIR of 33.69 (95% CI 32.02 to 35.43) in men and 30.00 (28.02 to 32.09) in women, corresponding to 483.72 and 96.00 excess cancer rates per 10 000 person-years, respectively. The risks decreased during longer follow-up periods but persisted over all follow-up periods for both sexes. For example, during 3–12 months of follow-up, the excess cancer rates per 10 000 person-years were 79.23 in men and 19.42 in women. After 5 years of follow-up, the excess cancer rates per 10 000 person-years were 9.17 in men and 2.50 in women. Table 4 shows an increased risk of bladder cancer across all age groups of men and women with cystitis, with suggested stronger associations in the younger age groups. A significant interaction can be seen between sex and cystitis in relation to bladder cancer risk, with men having a 1.07-fold (95% CI 1.03 to 1.12) higher relative cancer risk associated with cystitis than women. The interaction by sex is also shown across age groups; men had higher relative cancer risk than women aged 50–59 years (1.99-fold), 60–69 years (1.57-fold) and 70–79 years (1.23-fold). In contrast, men aged ≥80 years had a slightly lower (0.91-fold) relative cancer risk than women.

**Table 2:** Risks of subsequent urogenital cancer in men by follow-up time after acute cystitis

Follow-up times	O/E	SIR (95% CI)	Excess cancer rates per 10 000 person-years (95% CI)
Bladder cancer			
0 to <3 months	1523/45	33.69 (32.02 to 35.43)	483.72 (483.43 to 484.02)
3 to 12 months	932/148	6.32 (5.92 to 6.74)	79.23 (79.05 to 79.40)
>1 to 5 years	1255/582	2.16 (2.04 to 2.28)	17.87 (17.77 to 17.96)
>5 years	777/507	1.53 (1.43 to 1.64)	9.17 (9.06 to 9.28)
All	4487/1282	3.50 (3.40 to 3.60)	40.05 (39.99 to 40.11)
Kidney cancer			
0 to <3 months	174/15	11.28 (9.67 to 13.09)	51.91 (51.39 to 52.43)
3 to 12 months	132/51	2.61 (2.19 to 3.10)	8.23 (7.90 to 8.55)
>1 to 5 years	232/202	1.15 (1.01 to 1.31)	0.80 (0.61 to 0.99)
>5 years	180/172	1.05 (0.90 to 1.21)	0.26 (0.06 to 0.47)
All	718/440	1.63 (1.51 to 1.76)	3.47 (3.36 to 3.59)
Prostate cancer			
0 to <3 months	1961/278	7.05 (6.74 to 7.37)	550.88 (550.76 to 551.01)
3 to 12 months	1951/912	2.14 (2.04 to 2.24)	104.89 (104.82 to 104.97)
>1 to 5 years	3297/3604	0.91 (0.88 to 0.95)	−8.16 (−8.21 to −8.12)
>5 years	2563/2985	0.86 (0.83 to 0.89)	−14.34 (−14.39 to −14.29)
All	9772/7780	1.26 (1.23 to 1.28)	24.90 (24.87 to 24.93)

Excess cancer rates: observed urogenital cancer rates per 10 000 person-years compared with expected rate.

E, expected number of urogenital cancer cases; O, observed number of subsequent urogenital cancer cases in men with acute cystitis; SIR, standardised incidence ratio of the observed/expected urogenital cancers.

**Table 3:** Risks of subsequent urogenital cancer in women by follow-up time after acute cystitis

Follow-up times	O/E	SIR (95% CI)	Excess cancer rates per 10 000 person-years (95% CI)
Bladder cancer			
0 to <3 months	853/28	30.00 (28.02 to 32.09)	96.00 (95.63 to 96.37)
3 to 12 months	667/97	6.88 (6.37 to 7.42)	19.42 (19.20 to 19.63)
>1 to 5 years	1009/422	2.39 (2.24 to 2.54)	4.61 (4.49 to 4.72)
>5 years	806/476	1.69 (1.58 to 1.82)	2.50 (2.38 to 2.61)
All	3335/1023	3.26 (3.15 to 3.37)	7.77 (7.70 to 7.84)
Kidney cancer			
0 to <3 months	167/22	7.72 (6.60 to 8.99)	16.93 (16.48 to 17.37)
3 to 12 months	200/74	2.71 (2.35 to 3.11)	4.30 (4.03 to 4.56)
>1 to 5 years	427/322	1.33 (1.20 to 1.46)	0.83 (0.68 to 0.97)
>5 years	462/336	1.38 (1.25 to 1.51)	0.96 (0.82 to 1.10)
All	1256/753	1.67 (1.58 to 1.76)	1.69 (1.60 to 1.78)
Cervical cancer			
0 to <3 months	78/10	7.67 (6.06 to 9.58)	7.90 (7.24 to 8.55)
3 to 12 months	70/35	2.03 (1.58 to 2.56)	1.21 (0.80 to 1.62)
>1 to 5 years	166/146	1.14 (0.97 to 1.33)	0.16 (0.00 to 0.38)
>5 years	157/142	1.11 (0.94 to 1.30)	0.12 (0.00 to 0.34)
All	471/332	1.42 (1.29 to 1.55)	0.47 (0.33 to 0.61)
Endometrial cancer			
0 to <3 months	313/73	4.30 (3.84 to 4.81)	27.97 (27.72 to 28.23)
3 to 12 months	440/249	1.77 (1.61 to 1.94)	6.51 (6.35 to 6.66)
>1 to 5 years	1292/1078	1.20 (1.13 to 1.27)	1.68 (1.60 to 1.76)
>5 years	1361/1103	1.23 (1.17 to 1.30)	1.95 (1.87 to 2.03)
All	3406/2502	1.36 (1.32 to 1.41)	3.04 (2.99 to 3.09)
Ovarian cancer			
0 to <3 months	176/30	5.83 (5.00 to 6.76)	16.98 (16.59 to 17.36)
3 to 12 months	179/102	1.75 (1.51 to 2.03)	2.62 (2.38 to 2.86)
>1 to 5 years	463/418	1.11 (1.01 to 1.21)	0.35 (0.22 to 0.49)
>5 years	450/376	1.20 (1.09 to 1.31)	0.56 (0.43 to 0.70)
All	1268/926	1.37 (1.30 to 1.45)	1.15 (1.07 to 1.23)

Excess cancer rates: Observed urogenital cancer rates per 10,000 person-years compared to the expected rate.

E, expected number of urogenital cancer cases; O, observed number of subsequent urogenital cancer cases in men with acute cystitis; SIR, standardised incidence ratio of the observed/expected number of urogenital cancers.

**Table 4:** Risks of subsequent urinary tract cancers in men and women by age at acute cystitis diagnosis and interaction ratios by sex

Age (years)	Men		Women		IR (95% CI)
	O/E	SIR (95% CI)	O/E	SIR (95% CI)	
Bladder cancer					
50–59	28/2	18.30 (12.15 to 26.48)	30/3	9.20 (6.20–13.15)	1.99 (1.47 to 2.50)
60–69	569/86	6.61 (6.07 to 7.17)	408/97	4.21 (3.81–4.64)	1.57 (1.44 to 1.70)
70–79	1861/481	3.87 (3.69 to 4.05)	1297/412	3.15 (2.98–3.33)	1.23 (1.16 to 1.30)
≥80	2029/713	2.85 (2.72 to 2.97)	1600/512	3.13 (2.98–3.28)	0.91 (0.84 to 0.98)
All	4487/1282	3.50 (3.40 to 3.60)	3335/1023	3.26 (3.15–3.37)	1.07 (1.03 to 1.12)
Kidney cancer					
50–59	7/1	6.60 (2.62 to 13.68)	7/4	1.97 (0.78–4.09)	3.35 (2.30 to 4.40)
60–69	150/50	3.00 (2.54 to 3.52)	188/101	1.87 (1.61–2.15)	1.61 (1.39 to 1.82)
70–79	352/229	1.54 (1.38 to 1.71)	602/383	1.57 (1.45–1.70)	0.98 (0.85 to 1.11)
≥80	209/160	1.31 (1.13 to 1.50)	459/266	1.73 (1.57–1.89)	0.76 (0.59 to 0.92)
All	718/440	1.63 (1.51 to 1.76)	1256/753	1.67 (1.58–1.76)	0.98 (0.89 to 1.07)

E, expected number of urogenital cancer cases; IR, interaction ratio between the SIR in men and the SIR in women; O, observed number of subsequent urogenital cancer cases in men and women with acute cystitis; SIR, standardised incidence ratio of the observed/expected number of urogenital cancers.

For kidney cancer, the SIR was 1.63 (95% CI 1.51 to 1.76) in men with cystitis, corresponding to an excess cancer rate of 3.47 (3.36 to 3.59) per 10 000 person-years during follow-up. For women, the SIR was 1.67 (95% CI 1.58 to 1.76), corresponding to an excess cancer rate of 1.69 (1.60 to 1.78) per 10 000 person-years. The risks were particularly high within 3 months of follow-up, with SIRs for bladder cancer of 11.28 in men and 7.72 in women, corresponding to 51.91 and 16.93 excess rates per 10 000 person-years, respectively. The risks decreased with longer follow-up and persisted up to 5 years for men and >5 years for women; however, at a low level. Table 4 shows increased kidney cancer risk across all age groups of men and women with cystitis, with suggested stronger associations in younger age groups. We found no overall significant interaction between sex and cystitis in relation to kidney cancer risk. However, the relative kidney cancer risks associated with cystitis were significantly higher in men than women aged 50–59 years (3.35-fold) and 60–69 years (1.61-fold), whereas it was 0.76-fold lower in men than women aged ≥80 years.

### Prostate cancer

Table 2 shows that the SIR for prostate cancer was 1.26 (95% CI 1.23 to 1.28) in men with cystitis, corresponding to an excess cancer rate of 24.90 (24.87 to 24.93) per 10 000 person-years during follow-up. Within 12 months of follow-up, the risks were particularly high, with excess cancer rates per 10 000 person-years of 550.88 (95% CI 550.76 to 551.01) within the first 3 months of follow-up and 104.89 (104.82 to 104.97) within 3–12 months of follow-up. After 12 months of follow-up, the increased risk disappeared and the number of observed cases of prostate cancer was less than the expected (SIR<1). Table 5 shows increased prostate cancer risk across all age groups of men with cystitis, with an indication of stronger associations in younger age groups.

**Table 5:** Risks of subsequent prostate and gynaecological cancers by age at acute cystitis diagnosis

Age (years)	O/E	SIR (95% CI)	O/E	SIR (95% CI)
	Cervical cancer		Endometrial cancer	
50–59	5/3	1.94 (0.61 to 4.56)	20/16	1.27 (0.77 to 1.96)
60–69	58/46	1.26 (0.95 to 1.63)	481/393	1.23 (1.12 to 1.34)
70–79	188/137	1.37 (1.18 to 1.58)	1473/1134	1.30 (1.23 to 1.37)
≥80	220/146	1.50 (1.31 to 1.72)	1432/960	1.49 (1.42 to 1.57)
All	471/332	1.42 (1.29 to 1.55)	3406/2502	1.36 (1.32 to 1.41)
	Ovarian cancer		Prostate cancer	
50–59	17/10	1.65 (0.96 to 2.65)	36/10	3.64 (2.55 to 5.04)
60–69	267/186	1.44 (1.27 to 1.62)	1506/945	1.59 (1.51 to 1.68)
70–79	601/438	1.37 (1.27 to 1.49)	4488/3676	1.22 (1.19 to 1.26)
≥80	383/292	1.31 (1.18 to 1.45)	3742/3149	1.19 (1.15 to 1.23)
All	1268/925	1.37 (1.30 to 1.45)	9772/7780	1.26 (1.23 to 1.28)

E, expected number of urogenital cancer cases; O, observed number of subsequent urogenital cancer cases in men and women with acute cystitis; SIR, standardised incidence ratio of the observed/expected number of urogenital cancers.

### Gynaecological cancers

Table 5 shows that the risks of gynaecological cancers following cystitis were elevated across all age groups during follow-up. Table 3 shows that the risks were particularly high within the first 3 months of follow-up, with SIR of 7.67 (95% CI 6.06 to 9.58) for cervical cancer, 4.30 (3.84 to 4.81) for endometrial cancer, and 5.83 (5.00 to 6.76) for ovarian cancer; corresponding to 7.90 excess cervical, 27.97 excess endometrial, and 16.98 excess ovarian cancers per 10 000 person-years. The risks were reduced during 3–12 months of follow-up, but remained significant for all gynaecological cancer types, with excess cancer rates per 10 000 person-years of 6.51 for endometrial and 2.62 for ovarian cancers. The risks were further reduced after 12 months of follow-up, but remained significant for endometrial and ovarian cancer; however, at a low level.

## Discussion

In this population-based cohort study, which included 605 557 adults aged ≥50 years diagnosed with cystitis (91.3% in primary healthcare settings), cystitis preceded urogenital cancer across all age groups of men and women. The risks for urogenital cancers were highest within 3 months following the cystitis event, were particularly high for prostate and bladder cancers, and persisted across longer follow-up periods for most cancers. The results suggest that occult urogenital cancer should be considered in adults ≥50 years presenting with symptoms of cystitis, and that suspicion should be extra high for prostate and bladder cancer for which the risks were particularly high.

## Strengths and weaknesses

The main strengths of this study are the study size, long follow-up time, the use of several national registers of high quality, and the inclusion of population-based data from primary healthcare settings, where most acute cystitis events are diagnosed. All linkages between data were performed using a pseudonymised version of the personal identification number, allowing for virtually complete coverage of Sweden’s population, inclusion of potential confounders and practically no loss to follow-up.^21^ An important limitation of our study is that we did not have data on symptoms, urinary dipstik, or microbiological findings, and could thus not validate the cystitis diagnosis. Other researchers have, however, found significant bacteriuria to be present in 62–80% of patients clinically diagnosed with acute cystitis in Swedish primary healthcare.^38 39^ Misdiagnosis could be a partial explanation for our findings, particularly concerning the increased risk of urinary tract cancer shortly after a cystitis event, due to overlapping symptoms (eg, bladder cancer mistakenly diagnosed for cystitis).^5 7 17 18^ Moreover, because of the design of the study, we cannot rule out the risk of residual and unmeasured confounding, such as lifestyle factors (eg, smoking) and comorbidities (eg, obesity and undiagnosed diabetes mellitus). However, adjusting for these confounders would likely not be relevant for cancers occurring shortly after the cystitis diagnosis, as reversed causation is expected. Although residual/unmeasured confounding might exist for the cancers occurring later on during follow-up, we accounted for several comorbidities and sociodemographic factors, making it unlikely that such effects would be substantial. Nevertheless, smoking is a considerable risk factor for bladder and kidney cancers^40 41^ and may confound these estimates; yet, the current literature is inconsistent regarding the impact of smoking on urinary tract infections.^42 43^ Furthermore, the increased risks during longer follow-ups were much lower than those during shorter follow-ups. In addition, there is a possibility that adults with cystitis may be examined for cancers to a higher extent than those without the infection. However, any detection bias^44^ is likely lower in patients treated for cystitis in primary healthcare than in studies including patients admitted to hospitals with more severe infections,^1345^,^48^ who would be in closer contact with hospital systems, diagnostic procedures and cancer specialists. Lastly, if some patients with cystitis were not evaluated for cancer during follow-up, we could have missed cancers in the cystitis population, but since this delay, unfortunately, also occurs in the general population, we expect that any bias due to missed diagnoses is negligible.

### Comparison with other literature

Previous studies that have identified acute infections as markers of risk for subsequent cancer have encompassed less frequent but more severe systemic infections in hospital settings, for example, bacteraemia, pyelonephritis and sepsis,^1345^,^48^ while infections presenting to primary healthcare have not been studied as much. Our study adds to this literature with cystitis, primarily diagnosed in primary healthcare settings, as a potential clinical marker for occult urogenital cancer and an indicator for sustained subsequent risk of urogenital cancer. Previous studies on the associations between acute cystitis and urogenital cancer have, to our knowledge, not included primary healthcare data and have been limited to small sample sizes and follow-up. For example, a systematic review and meta-analysis of 16 studies concluded that exposure to UTI favours increased subsequent risk of bladder cancer (risk ratio 1.33, 95% CI 1.14 to 1.55),^14^ which is comparable to our findings (SIR 3.50 in men and 3.26 in women). However, the results from the meta-analysis were inconclusive when excluding studies at high risk of bias.^14^ A previous study from Taiwan,^15^ based on insurance data (1998–2011), examined the association between cystitis and prostate cancer and found similar results to ours. In that study, the authors included 9347 men with cystitis for comparison with 4:1 age-matched controls without cystitis. The study found that the risk of prostate cancer was 46% higher in the cystitis cohort compared with those without cystitis, which is aligned with our findings (SIR 1.26). Another similar study, based on insurance data (2009–2013) from Taiwan,^16^ found similar associations between cystitis and urogenital cancer. In that study, the authors included 38 084 people with cystitis and 76 168 propensity score-matched people in a control group. The authors found that risks for urogenital cancer were 32% and 21% higher in men and women with cystitis compared with men and women without cystitis, respectively. Moreover, the study also found similar increases in risks for specific cancers to our findings; for example, the adjusted hazard ratios for subsequent bladder and kidney cancer were 28.60 (95% CI 6.80 to 120.28) and 3.85 (95% CI 1.42 to 10.42) for kidney cancer in men ≥65 years of age.

There are several possible explanations behind these and our findings. It is plausible that urogenital cancer, and perhaps even precancerous changes in the urogenital organ, might increase the risk of cystitis because of compromised urinary tract and host defence.^3^ Moreover, it is possible that certain occult urogenital cancers, especially urinary tract cancers, could present symptoms similar to those of cystitis,^5 7 17 18^ which might explain the particularly high risk of subsequent urogenital cancer shortly after the cystitis event. Altogether, the alignment between the results of our nationwide study and previous smaller studies from other countries, as well as plausible mechanisms, strengthens the generalisability and robustness of the identified associations between cystitis and subsequent urogenital cancer risks.

### Implications and further research

The present study adds to the accumulating evidence of infections as markers of increased cancer risk.^1345^,^48^ For clinicians, the findings indicate that acute cystitis could be a clinical marker for urogenital cancer (at least when no other cause is obvious), and particularly for occult urogenital cancer, as the risks for cancers were highest within 3 months of cystitis diagnosis. Although the risks for urogenital cancers were attenuated over time, the risks remained high up to 12 months after acute cystitis for most urogenital cancers, suggesting a possibility for earlier cancer detection. Beyond 1 year of follow-up from cystitis diagnosis, the elevated risks of urogenital cancers decreased further for most cancers, with excess cancer rates below 1 per 10 000 person-years for most cancers, except bladder and endometrial cancers. This pattern may reflect a compensatory deficit of cancer diagnoses after an increased detection rate in the first 12 months after cystitis, indicating that follow-up beyond 1 year might have limited cost benefit. For healthcare, the implication is especially important for primary healthcare, as most cystitis cases are diagnosed and many forms of cancer present in this setting.^17 18^ Additionally, our findings imply that cystitis precedes urogenital cancer differently across sex and age groups, indicating that the clinical relevance of cystitis as a potential marker for urogenital cancers may vary across populations. For policymakers, it remains to be determined whether the sustained long-term subsequent risks of urogenital cancers following cystitis could be used to help earlier detection or even prevention of urogenital cancers. For example, precancerous changes in the urogenital system may facilitate cystitis by compromising the urinary tract. Lastly, considering that haematuria can be a symptom of acute cystitis and urinary tract cancers,^3 18^ future studies could also examine whether those with concomitant cystitis and haematuria have an increased risk of subsequent urinary tract cancers compared with those presenting with either cystitis or haematuria.

In conclusion, this population-based cohort study found that acute cystitis is a common infection among adults aged ≥50 years, often diagnosed in primary healthcare settings, and can precede urogenital cancers. The cancer risks were particularly high within 3 months after acute cystitis, suggesting that acute cystitis might be a useful clinical marker for urogenital cancer, especially for bladder and prostate cancer. The persistent risk beyond 3 months might present an opportunity for earlier detection of urogenital cancer, but this warrants further research including health economic evaluations.

## Supplementary material

10.1136/bmjph-2024-002495 online supplemental file 1

10.1136/bmjph-2024-002495 online supplemental file 2

10.1136/bmjph-2024-002495 online supplemental file 3

## Data Availability

Data may be obtained from a third party and are not publicly available.

## References

[1] Medina M, Castillo-Pino E (2019). An introduction to the epidemiology and burden of urinary tract infections. Ther Adv Urol.

[2] Stamm WE, Norrby SR (2001). Urinary tract infections: disease panorama and challenges. J Infect Dis.

[3] Flores-Mireles AL, Walker JN, Caparon M (2015). Urinary tract infections: epidemiology, mechanisms of infection and treatment options. Nat Rev Microbiol.

[4] Jansåker F, Li X, Sundquist K (2021). Sociodemographic factors and uncomplicated cystitis in women aged 15-50 years: a nationwide Swedish cohort registry study (1997-2018). *Lancet Reg Health Eur*.

[5] Nicolle LE (2008). Uncomplicated urinary tract infection in adults including uncomplicated pyelonephritis. Urol Clin North Am.

[6] Sundquist K, Li X, Jansåker F (2021). Sociodemographic factors and uncomplicated pyelonephritis in women aged 15-50 years: a nationwide Swedish cohort register study (1997-2018). Int J Infect Dis.

[7] Wagenlehner FME, Bjerklund Johansen TE, Cai T (2020). Epidemiology, definition and treatment of complicated urinary tract infections. Nat Rev Urol.

[8] Griebling TL (2005). Urologic diseases in america project: trends in resource use for urinary tract infections in men. J Urol.

[9] Deltourbe L, Lacerda Mariano L, Hreha TN (2022). The impact of biological sex on diseases of the urinary tract. Mucosal Immunol.

[10] Cho NH, Shaw JE, Karuranga S (2018). IDF Diabetes Atlas: Global estimates of diabetes prevalence for 2017 and projections for 2045. Diabetes Res Clin Pract.

[11] White MC, Holman DM, Boehm JE (2014). Age and cancer risk: a potentially modifiable relationship. Am J Prev Med.

[12] Torre LA, Bray F, Siegel RL (2015). Global cancer statistics, 2012. CA Cancer J Clin.

[13] Søgaard KK, Veres K, Nørgaard M (2019). Pyelonephritis in persons after age 50 as a clinical marker of urogenital cancer. Clin Microbiol Infect.

[14] Bayne CE, Farah D, Herbst KW (2018). Role of urinary tract infection in bladder cancer: a systematic review and meta-analysis. World J Urol.

[15] Fan C-Y, Huang W-Y, Lin K-T (2017). Lower Urinary Tract Infection and Subsequent Risk of Prostate Cancer: A Nationwide Population-Based Cohort Study. PLoS One.

[16] Huang C-H, Chou Y-H, Yeh H-W (2019). Risk of Cancer after Lower Urinary Tract Infection: A Population-Based Cohort Study. Int J Environ Res Public Health.

[17] Hamilton W (2010). Cancer diagnosis in primary care. Br J Gen Pract.

[18] Holtedahl K, Borgquist L, Donker GA (2023). Symptoms and signs of urogenital cancer in primary care. *BMC Prim Care*.

[19] SCB Official statistics of sweden. Population and population changes 1749–2023. https://www.scb.se/en/finding-statistics/statistics-by-subject-area/population/population-composition/population-statistics/pong/tables-and-graphs/population-statistics---summary/population-and-population-changes/.

[20] Ludvigsson JF, Bergman D, Lundgren CI (2025). The healthcare system in Sweden. Eur J Epidemiol.

[21] Ludvigsson JF, Otterblad-Olausson P, Pettersson BU (2009). The Swedish personal identity number: possibilities and pitfalls in healthcare and medical research. Eur J Epidemiol.

[22] Barlow L, Westergren K, Holmberg L (2009). The completeness of the Swedish Cancer Register: a sample survey for year 1998. Acta Oncol.

[23] Ludvigsson JF, Andersson E, Ekbom A (2011). External review and validation of the Swedish national inpatient register. BMC Public Health.

[24] Ludvigsson JF, Almqvist C, Bonamy A-KE (2016). Registers of the Swedish total population and their use in medical research. Eur J Epidemiol.

[25] Sundquist J, Ohlsson H, Sundquist K (2017). Common adult psychiatric disorders in Swedish primary care where most mental health patients are treated. BMC Psychiatry.

[26] Bengtsen MB, Farkas DK, Borre M (2021). Acute urinary retention and risk of cancer: population based Danish cohort study. BMJ.

[27] Clegg LX, Reichman ME, Miller BA (2009). Impact of socioeconomic status on cancer incidence and stage at diagnosis: selected findings from the surveillance, epidemiology, and end results: National Longitudinal Mortality Study. Cancer Causes Control.

[28] Hemminki K, Li X, Czene K (2002). Cancer risks in first-generation immigrants to Sweden. Int J Cancer.

[29] Holt RIG, Cockram CS, Ma RCW (2024). Diabetes and infection: review of the epidemiology, mechanisms and principles of treatment. Diabetologia.

[30] Hemminki K, Li X, Sundquist J (2010). Risk of cancer following hospitalization for type 2 diabetes. Oncologist.

[31] Christiansen CF, Onega T, Sværke C (2014). Risk and prognosis of cancer in patients with nephrotic syndrome. Am J Med.

[32] Tandogdu Z, Cai T, Koves B (2016). Urinary Tract Infections in Immunocompromised Patients with Diabetes, Chronic Kidney Disease, and Kidney Transplant. Eur Urol Focus.

[33] Mortaz E, Tabarsi P, Mansouri D (2016). Cancers Related to Immunodeficiencies: Update and Perspectives. Front Immunol.

[34] Jansåker F, Li X, Knudsen JD (2022). The association between common urogenital infections and cervical neoplasia - A nationwide cohort study of over four million women (2002-2018). *Lancet Reg Health Eur*.

[35] Mihalopoulos M, Yaghoubian A, Razdan S (2022). Understanding the link between kidney stones and cancers of the upper urinary tract and bladder. Am J Clin Exp Urol.

[36] Miah S, Catto J (2014). BPH and prostate cancer risk. Indian J Urol.

[37] Estève J, Benhamou E, Raymond L (1994). Statistical methods in cancer research. Volume IV. Descriptive epidemiology. IARC Sci Publ.

[38] Kornfält Isberg H, Hedin K, Melander E (2020). Different antibiotic regimes in men diagnosed with lower urinary tract infection - a retrospective register-based study. Scand J Prim Health Care.

[39] Kornfält Isberg H, Melander E, Hedin K (2019). Uncomplicated urinary tract infections in Swedish primary care; etiology, resistance and treatment. BMC Infect Dis.

[40] Mori K, Mostafaei H, Abufaraj M (2020). Smoking and bladder cancer: review of the recent literature. Curr Opin Urol.

[41] Liu X, Peveri G, Bosetti C (2019). Dose-response relationships between cigarette smoking and kidney cancer: A systematic review and meta-analysis. Crit Rev Oncol Hematol.

[42] Wallin HP, Gissler M, Korhonen PE (2023). New insights into smoking and urinary tract infections during pregnancy using pregnancy-pair design: A population-based register study. Acta Obstet Gynecol Scand.

[43] Alnaif B, Drutz HP (2001). The association of smoking with vaginal flora, urinary tract infection, pelvic floor prolapse, and post-void residual volumes. J Low Genit Tract Dis.

[44] Johnson JA, Bowker SL, Richardson K (2011). Time-varying incidence of cancer after the onset of type 2 diabetes: evidence of potential detection bias. Diabetologia.

[45] Søgaard KK, Farkas DK, Søgaard M (2017). Gram-negative bacteremia as a clinical marker of occult malignancy. J Infect.

[46] Gotland N, Uhre ML, Sandholdt H (2020). Increased risk of incident primary cancer after Staphylococcus aureus bacteremia: A matched cohort study. Medicine (Baltimore).

[47] Justesen US, Nielsen SL, Jensen TG (2022). Bacteremia With Anaerobic Bacteria and Association With Colorectal Cancer: A Population-based Cohort Study. Clin Infect Dis.

[48] Hästbacka J, But A, Strandberg G (2023). Risk of malignant disease in 1-year sepsis survivors, a registry-based nationwide follow-up study. Crit Care.

