# Supplementary material for: Acute cystitis and subsequent risk of urogenital cancer: a national cohort study from Sweden
Source: BMJ Public Health. 2025 Sep 16;3(2):e002495. doi: 10.1136/bmjph-2024-002495 (PMC12443172; doi:10.1136/bmjph-2024-002495)
Supplement: online supplemental file 1 [file bmjph-3-2-s001.docx]

**Statistical formulas**

The formula used for calculating the standardized incidence ratios (SIRs) was:

$$SIR= \frac{\sum_{j=1}^{J} O_{j}}{\sum_{i=1}^{J} n_{j}{}_{j}^{*}}=\frac{O}{E^{*}}$$

The 95% confidence intervals (CIs) of the SIRs were calculated assuming a Poisson distribution were calculated with the following formula:

$$\frac{\left( \frac{1.96}{2}\pm\sqrt{O} \right)^{2}}{E^{*}}$$

The formula used for calculating the 95% CI of the excess cancer rates per 10,000 person-years were:

$$\left( \mathrm{Excess}rate\pm1.96*\sqrt{\frac{1}{O}+\frac{1}{E^{*}}} \right)$$
