# Supplementary material for: Acute cystitis and subsequent risk of urogenital cancer: a national cohort study from Sweden
Source: BMJ Public Health. 2025 Sep 16;3(2):e002495. doi: 10.1136/bmjph-2024-002495 (PMC12443172; doi:10.1136/bmjph-2024-002495)
Supplement: online supplemental file 2 [file bmjph-3-2-s002.docx]

**Supplementary tables**

**Table S1.** Sociodemographic characteristics and comorbidities of the total population of adults aged ≥50 years (n= 3,557,582) without prior diagnosis of urogenital cancer

Page 2.

**Table S2.** Number of men and women aged ≥50 years diagnosed with cystitis (lower urinary tract infection), setting, and mean age at diagnosis during follow-up (1997–2018)

Page 3.

**Table S3.** Number of men and women aged ≥50 years diagnosed with urogenital cancers and mean age at diagnosis during follow-up (1997–2018)

Page 4.

| **Table S1.** Sociodemographic characteristics and comorbidities of the total population of adults aged ≥50 years (n= 3,557,582) without prior diagnosis of urogenital cancer | | | | |
| --- | --- | --- | --- | --- |
|  | | No. | | % |
| **Sex** | |  | |  |
| Male | | 1,668,371 | | 46.9 |
| Female | | 1,889,211 | | 53.1 |
| **Educational Level** | |  | |  |
| <12 years | | 2,600,034 | | 73.1 |
| ≥12 years | | 957,548 | | 26.9 |
| **Region of residence** | |  | |  |
| Large cities | | 1,932,662 | | 54.3 |
| Other | | 1,624,920 | | 45.7 |
| **Country of origin** | |  | |  |
| Born in Sweden | | 3,043,254 | | 85.5 |
| Born outside of Sweden | | 514,328 | | 14.5 |
| **Comorbidities** |  | |  |  |
| Diabetes mellitus | | 509,855 | | 14.3 |
| Immunodeficiency disorders | | 10,677 | | 0.3 |
| Nephritic or nephrotic syndrome | | 114,749 | | 3.2 |
| Urolithiasis | | 107,835 | | 3.0 |
| Non-malignant prostate diseases | | 377,895 | | 10.6 |
| Bacterial vaginosis | | 9923 | | 0.3 |
| Non-malignant prostate diseases include benign prostate hyperplasia and prostatitis. The proportion of men with non-malignant prostate diseases was 22.7% and the proportion of women with bacterial vaginosis was 0.5%. Sociodemographic factors were assessed at baseline (1997) and comorbidities were identified during the study period. Data sources: Total Population Register, National Patient Register, and primary healthcare data. | | | | |

| **Table S2.**  Number of men and women aged ≥50 years diagnosed with acute cystitis (lower urinary tract infection), setting, and mean age at diagnosis during follow-up (1997–2018) | | | | |
| --- | --- | --- | --- | --- |
|  | ICD-10 code | No. | % | Mean age (± SD) (years) |
| **Cystitis cohort** | N30 | 605,557 |  | 75.9 ± 9.2 |
| Out-patient setting (National Patient Register) | N30 | 52,883 | 8.7 | 74.9 ± 9.4 |
| Primary healthcare setting | N30 | 552,674 | 91.3 | 76.0 ± 9.2 |
| ICD-10: The 10^th^ revision of the International Classification of Diseases. | | | | |

| **Table S3.** Number of men and women aged ≥50 years diagnosed with urogenital cancers and mean age at diagnosis during follow-up (1997–2018) | | | | | |
| --- | --- | --- | --- | --- | --- |
|  | ICD-7 codes | No. | % |  | Mean age ± SD (years) |
|  |  |  |  |  |  |
| **Total number of urogenital cancer cases** |  | 257,026 |  |  | 73.52 ± 8.26 |
| Cervical cancer | 171 | 3633 | 1.4 |  | 73.38 ± 9.69 |
| Endometrial cancer | 172, 174 | 24,672 | 9.6 |  | 73.16 ± 9.09 |
| Ovarian cancer | 175 | 10,787 | 4.2 |  | 71.37 ± 9.08 |
| Prostate cancer | 177 | 158,732 | 61.8 |  | 73.26 ± 7.81 |
| Kidney cancer | 180 | 16,962 | 6.6 |  | 72.65 ± 8.14 |
| Bladder cancer | 181 | 42,240 | 16.4 |  | 75.60 ± 8.65 |
|  |  |  |  |  |  |
| **Subsequent cancers cases after cystitis** |  | 24,137 |  |  | 76.90 ± 7.72 |
| Cervical cancer | 171 | 463 | 1.9 |  | 77.65 ± 7.87 |
| Endometrial cancer | 172, 174 | 3327 | 13.8 |  | 77.20 ± 7.83 |
| Ovarian cancer | 175 | 1254 | 5.2 |  | 74.89 ± 7.61 |
| Prostate cancer | 177 | 9537 | 39.5 |  | 76.51 ± 7.48 |
| Kidney cancer | 180 | 1923 | 8.0 |  | 75.63 ± 7.18 |
| Bladder cancer | 181 | 7633 | 31.6 |  | 77.87 ± 7.95 |
| ICD-7: The 7^th^ revision of the International Classification of Diseases. Data source: Swedish Cancer Register. | | | | | |
