# Supplementary material for: Acute cystitis and subsequent risk of urogenital cancer: a national cohort study from Sweden
Source: BMJ Public Health. 2025 Sep 16;3(2):e002495. doi: 10.1136/bmjph-2024-002495 (PMC12443172; doi:10.1136/bmjph-2024-002495)
Supplement: online supplemental file 3 [file bmjph-3-2-s003.docx]

**Figure S1.** Incidence rates per 10,000 person-years (y-axis) of bladder cancer in men and women aged ≥50 years, with or without cystitis, by age groups (x-axis)

**Figure S2.** Incidence rates per 10,000 person-years (y-axis) of kidney cancer in men and women aged ≥50 years, with or without cystitis, by age groups (x-axis)

**Figure S3.** Incidence rates per 10,000 person-years (y-axis) of prostate cancer in men aged ≥50 years, with or without cystitis, by age groups (x-axis)

**Figure S4.** Incidence rates per 10,000 person-years (y-axis) of cervical cancer in women aged ≥50 years, with or without cystitis, by age groups (x-axis)

**Figure S5.** Incidence rates per 10,000 person-years (y-axis) of endometrial cancer in women aged ≥50 years, with or without cystitis, by age groups (x-axis)

**Figure S6.** Incidence rates per 10,000 person-years (y-axis) of ovarian cancer in women aged ≥50 years, with or without cystitis, by age groups (x-axis)
